# Machine Learning-Based Soft Sensor for Real-Time Wire Bow Prediction in Diamond Multi-Wire Sawing

**DOI:** 10.3390/s26061875

**Published:** 2026-03-16

**Authors:** Xiangyu Zhao, Hua Liu, Jie Yang, Liang Zhu, Heng Li, Lemiao Qiu, Shuyou Zhang

**Affiliations:** 1 School of Mechanical Engineering, Zhejiang University, 866 Yuhangtang Rd, Hangzhou 310058, China; zhaoxiangyu@jsjd.cc (X.Z.);; 2 Zhejiang Qiushi Semiconductor Equipment Co., Ltd., 500 Shunda Rd, Hangzhou 311100, China

**Keywords:** diamond multi-wire sawing, wire bow, soft sensor, machine learning, XGBoost

## Abstract

Real-time monitoring of wire bow is critical for ensuring wafer quality and preventing wire breakage in diamond multi-wire sawing (MWS). However, the deployment physical sensors in industrial MWS environments is hindered by severe sludge contamination, limited installation space, and high maintenance costs. To address these challenges, this paper proposes a novel data-driven soft sensor framework utilizing machine learning methods to predict wire bow based on readily accessible process data. A feature engineering pipeline, combining variance thresholding and correlation analysis, is established to identify key process variables. Subsequently, six representative ML algorithms are systematically evaluated, with eXtreme Gradient Boosting (XGBoost) optimized via two-stage hyperparameter optimization emerging as the superior model. Experimental results from an industrial MWS machine demonstrate that the proposed model achieves a coefficient of determination ($R^{2}$) of 0.992 and a mean absolute error (MAE) of 0.116 mm. Furthermore, the prediction is also extended to spatially distributed positions (head, middle, and tail) of the wire web. Finally, SHAP (SHapley Additive exPlanations) is utilized to elucidate the mechanical dependencies. This work provides a reliable and low-cost solution for wire bow monitoring during the MWS process.

## 1. Introduction

Diamond multi-wire sawing (MWS) technology is a pivotal process for wafer fabrication in the photovoltaic (PV) and semiconductor industries [1,2,3]. This technique employs a multi-wire web embedded with diamond particles to cut silicon ingots into thin wafers via reciprocating motion [4]. During the cutting process, the silicon brick exerts a normal force on the wire web, inducing a deflection phenomenon commonly referred to as “wire bow” [5]. The wire bow is essential for generating the necessary cutting force and serves as a direct indicator of the diamond wire’s cutting capability. Moreover, it is closely associated with the wafer surface quality [6]. Driven by the imperative for higher material utilization and production efficiency, the industry has shifted toward finer-diameter wires (<35 μm) and more aggressive process parameters [7]. This trend exacerbates the larger wire bow. Excessive wire bow not only intensifies wire saw marks and warpage but also elevates the risk of wire breakage, resulting in process interruptions and material yield loss [8]. Consequently, precise real-time monitoring of the wire bow is of great importance, and it lays the foundation for early breakage detection and the adaptive regulation of cutting parameters.

Wire bow measurement and monitoring have been the subject of extensive research. Clark et al. [9] employed non-contact capacitive sensors to quantify the wire bow angle in rocking-motion diamond wire saw machines. Qiu et al. [10] investigated the formation mechanism of the wire bow using an eddy current sensor. Coustier et al. [11] developed a real-time monitoring instrument comprising 12 fixed eddy current sensors to elucidate the variation laws of wire bow across different positions during industrial MWS. Optical techniques represent another prevalent approach, consisting of high-speed imaging and laser profiling. For instance, Liu et al. [12] and Guo et al. [8] utilized high-definition digital cameras to capture wire morphology and analyzed the correlation between varying process parameters, slicing depths, and wire bow. Qin et al. [13] quantified the deflection angle of the diamond wire in real time by tracking its positional changes via light projection techniques. Zhang et al. [14] proposed a dynamic scanning system using a line laser sensor to monitor the wire bow for the stone cutting applications.

However, the deployment of such sensors in industrial environments faces substantial challenges. Firstly, the internal environment of the MWS equipment is extremely harsh and dirty, replete with spattered silicon sludge and cutting fluid during the sawing process of silicon bricks [15,16]. This contamination severely interferes with signal integrity, particularly for optical sensors. Consequently, frequent cleaning and re-calibration are necessary to ensure data fidelity, which significantly undermines equipment availability. Secondly, some sensors (notably eddy current sensors) suffer from limited measurement ranges [17], necessitating close proximity to the wire web. This constraint not only fails to accommodate large wire bow scenarios but also heightens the risk of sensor damage from high-speed wires. Finally, the relatively high cost of these high-precision sensors substantially increases the overall system investment.

Soft sensor technology, which estimates the variables that are difficult to measure directly using more easily measurable ones, has been widely validated in various industrial processes [18,19,20]. A common category is the model-driven soft sensor, typically constructed upon first-principle (mechanistic) models. Numerous studies have proposed mechanistic models to explain the formation mechanisms and governing factors relevant to wire bow in wire saw processes [10,21,22,23,24,25,26]. Nevertheless, these models are seldom implemented in practical production for real-time prediction. This limited applicability is due to their heavy reliance on idealized assumptions (e.g., uniform cutting force distribution) and the requirement for parameters that are difficult to monitor online, such as the cutting force. In addition, the highly interdependent and non-linear nature of the factors influencing wire bow renders mechanistic modeling insufficient for complex industrial wire saw scenarios.

Data-driven models based on machine learning (ML) methods have emerged as a prevalent alternative to address these challenges [27,28,29,30]. While Zhang et al. [14] pioneered the application of ML to predict wire bow in single-wire sawing for stone, research focusing on the MWS process remains scarce. Compared to single-wire systems, the MWS process involves intricate inter-wire interactions, leading to more complex mechanisms of wire bow formation. Furthermore, wire bow characteristics exhibit significant spatial variability across different positions within the wire web [11].

To bridge this research gap and address the complexities of MWS, this study proposes an ML-based soft sensor for wire bow prediction in the MWS of silicon ingots. The core contribution of this research lies in pioneering a comprehensive data-driven solution for the complex MWS scenario, providing a practical reference for deploying soft sensors in the wafer fabrication process. By utilizing historical industrial data, this data-driven approach directly captures the non-linear mapping between multi-dimensional process parameters and wire bow, effectively circumventing the limitations of the mechanistic assumptions. This research systematically evaluates several representative ML algorithms, including Support Vector Regression (SVR), Multi-layer Perceptron (MLP), Decision Tree Regression (DTR), Random Forest (RF), Gradient Boosting (GB) and eXtreme Gradient Boosting (XGBoost). Experimental results demonstrate that the proposed ML framework achieves high-precision estimation, providing a precise, cost-effective, and real-time solution for wire bow monitoring in the MWS process.

The main contributions of this paper are summarized as follows:

- An application-oriented soft sensor framework is proposed for real-time wire bow prediction in the MWS process. This data-driven approach establishes a novel application scenario, effectively overcoming the severe limitations associated with physical sensors in harsh manufacturing environments, and provides a valuable reference for implementing intelligent monitoring in MWS.
- A systematic optimization and evaluation of machine learning models for wire bow estimation is presented. Through two-stage hyperparameter optimization, the XGBoost model is identified as the optimal solution, demonstrating superior generalization capability ($R^{2}=0.992$) and stability compared to the other ML algorithms (e.g., SVR, MLP). Notably, the model exhibits accurate predictive performance not only for the global mean wire bow but also across spatially distributed positions (head, middle, and tail) of the wire web.
- An in-depth analysis of the physical interpretability of the model is conducted. The study utilizes SHAP values to quantify the dominant influence of spindle and feed torques, thereby validating the consistency between the data-driven model and the physical cutting mechanisms.

## 2. Methodology

This section is organized as follows. First, the MWS equipment is introduced, followed by the definition and formation mechanisms of the wire bow. Second, the online measurement system for wire bow data acquisition is described, along with its practical limitations in industrial production, which underscores the necessity of developing a soft sensor. Subsequently, the machine learning algorithms and hyperparameter optimization methods employed for soft sensor construction are detailed. Finally, the overall framework and workflow of the proposed soft sensor modeling process are presented.

### 2.1. Wire Bow in MWS

#### 2.1.1. Definition of Wire Bow

**Figure 1:**
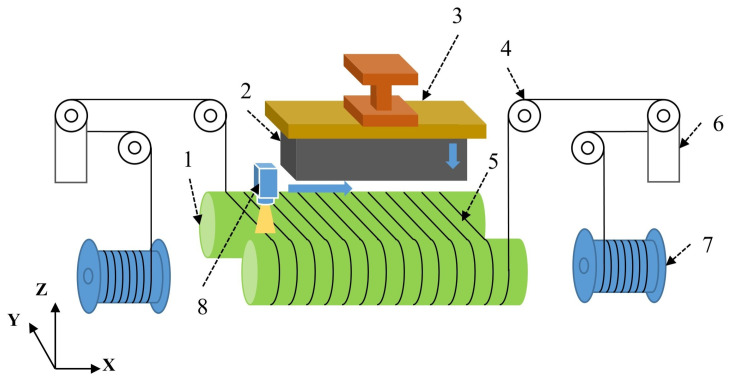
Schematic diagram of the multi-wire sawing machine: 1. guide roller; 2. silicon brick; 3. feeding mechanism; 4. wire wheels; 5. diamond wire web; 6. tension arm; 7. wire-in/out spool; 8. wire saw sensor (laser profiler).

The core structure of the MWS machine is illustrated in Figure 1. The system primarily comprises a pair of grooved guide rollers, tension arms, the feeding mechanism, wire wheels, and wire-in/out spools. The diamond wire is released from the wire-in spool to the wire-out one, passes through the wire wheels and the guide rollers, and is then wound around the grooved rollers to form a dense, parallel wire web. During the cutting process, the guide rollers are driven by a high-power spindle motor to achieve high-speed reciprocating motion. Simultaneously, the silicon ingot is pressed into the wire web by the vertical feeding mechanism. This synchronized motion enables the simultaneous slicing of the silicon ingot into thousands of thin wafers through the back-and-forth motion of the wire web.

**Figure 2:**
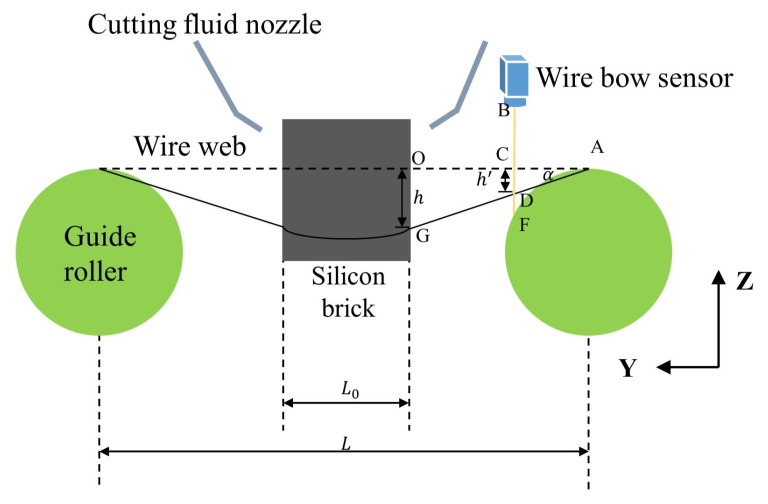
Diagram of wire bow.

The diagram of wire bow is illustrated in Figure 2. During the cutting process, the wire web undergoes bending induced by the downward feeding pressure of the silicon ingot. For the purpose of quantitative analysis in this study, the vertical displacement height of the wire web, denoted as *h*, is adopted as the representative parameter for the wire bow. In practical industrial production, the wire sawing process is frequently interrupted at scheduled intervals to manually monitor the value of *h*. If the value exceeds a predefined threshold, process parameters must be adjusted immediately to prevent wire breakage and wafer quality degradation.

#### 2.1.2. Formation Mechanism of Wire Bow

Liedke et al. [22] provided the following expression for the wire bow *h*:

(1) $$\begin{array}{c} \dot{h}+K\left(t\right)h=v_{f}\left(t\right) \end{array}$$

(2) $$\begin{array}{c} K\left(t\right)=\frac{4Tk_{p}v_{s}\left(t\right)}{L_{0}d_{w}\left(L-L_{0}\right)} \end{array}$$

where *h* represents the wire bow deflection, and $\dot{h}$ is its time derivative. $v_{f}\left(t\right)$ and $v_{s}\left(t\right)$ denote the feed rate of silicon brick and the absolute wire velocity, respectively. Regarding the geometric and physical parameters: $L_{0}$ is the silicon ingot width, *L* represents the spacing between guide rollers, $d_{w}$ is the wire diameter, and *T* indicates the tension of the wire. The parameter $k_{p}$ is the Preston coefficient, characterizing the material removal rate. Under the assumption of some time-invariant process parameters and a zero initial deflection, Equation (3) is derived to describe the temporal evolution of the wire bow *h*:

(3) $$h\left(t\right)=\frac{L_{0}d_{w}\left(L-L_{0}\right)}{4Tk_{p}}\frac{v_{f}}{v_{s}}\left(1-e^{-\frac{4Tk_{p}v_{s}t}{L_{0}d_{w}\left(L-L_{0}\right)}}\right)$$

Based on Equation (3), the key influencing factors of the wire bow are *T*, $v_{s}$, $v_{f}$, $k_{p}$, and $d_{w}$, among others. The general expression is given by:

(4) $$h=f(T,v_{s},v_{f},k_{p},d_{w},\ldots )$$

**Figure 3:**
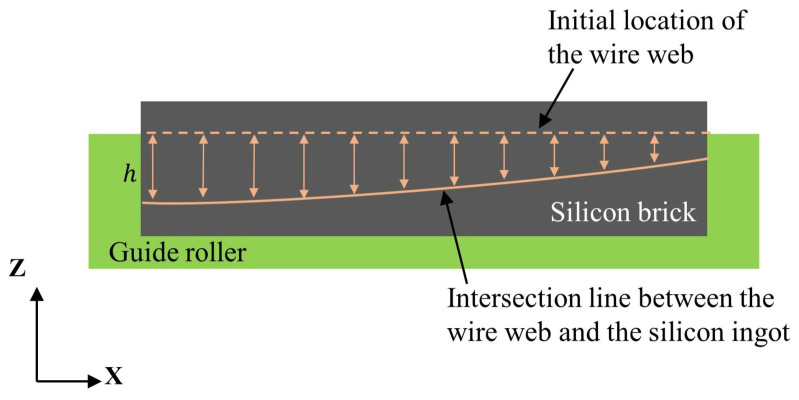
Schematic diagram of wire bow *h* variation along the spindle axis (*x*-axis).

Generally, the non-uniform wire wear at different positions implies that the Preston coefficient $k_{p}$ and the wire diameter $d_{w}$ cannot be treated as a constant [11]. Furthermore, there is a variation in tension *T* along the spindle axis. Consequently, at any given instant, the wire bow exhibits spatial variation along the axial direction, forming the wire bow profile illustrated in Figure 3.

It should be noted that while mechanistic models successfully identify the influencing factors of the wire bow, they are difficult to implement in real-time soft sensing. This is because such models rely on numerous idealized assumptions primarily. Furthermore, critical parameters, such as the Preston coefficient $k_{p}$ and the wire tension at various positions, are difficult to measure online in real-time.

### 2.2. Wire Bow Monitoring System

**Figure 4:**
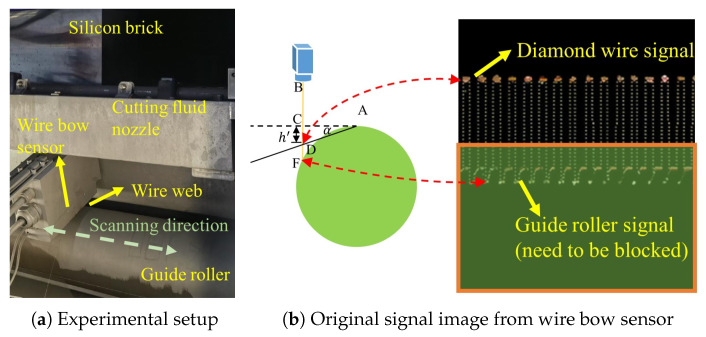
Experimental setup and signal acquisition of the wire bow monitoring system.

To provide the wire bow data for the soft sensor modeling, an online monitoring wire bow system is developed using a line laser profiler, as shown in Figure 4a. The sensor is oriented normally to the wire web. Due to the structural constraints imposed by the cutting fluid nozzle, the sensor is positioned at a distance from the silicon ingot. The sensor scans across the wire web along the spindle axis to acquire spatial distance profiles at various positions.

As illustrated in Figure 2, the procedure for extracting the wire bow *h* is summarized as follows: (1) The sensor’s range spans both the wire web and the guide roller. The laser beam intersects the wire web and the guide roller at points *D* and *F*, respectively. Therefore, the signals from the guide roller must be filtered out, as shown in Figure 4b, to extract the wire web data. (2) Prior to the cutting process, an initial scan of the wire web is performed to obtain the distance *BC*, which is defined as the reference height; (3) During the sawing process, one full scan of the laser profiler is acquired at each wire reversal. The sensor monitors the distance *BD* continuously; (4) The vertical displacement at the laser incidence point *C* is then calculated by the difference: $h^{\prime }=BD-BC$; (5) Based on the principle of geometric similarity, the local displacement $h^{\prime }$ is mapped to the actual wire bow *h*.

### 2.3. Limitations of the Physical Monitoring System

Although the monitoring system based on the laser profiler can effectively captures the dynamic evolution of the wire bow, the performance of the sensor module degrades progressively as the number of cutting runs increases. This physical monitoring approach reveals several critical limitations:

- **Lens contamination:** Despite the implementation of hermetically sealed protective housings for the line laser sensor shown in Figure 4a, the lens contamination due to the silicon sludge remains inevitable after a limited number of runs. To ensure the data accuracy, the sensor must be periodically disassembled for thorough cleaning.
- **Stability degradation of the scanning guideway:** The silicon sludge not only fouls the optical lens but also infiltrates and accumulates along the scanning guideway. This leads to stochastic trembling during sensor scanning, which is the primary source of measurement error in the laser profiler [31]. Simultaneously, this may render the previously blocked signals of the main roller, as illustrated in Figure 4b, to reappear.
- **Reference baseline drift:** The reference height *BC* is conventionally assumed to be constant. However, in actual scenarios, the deformations of the guide rollers can cause a drift in the initial baseline. Therefore, frequent re-calibration is necessary, which severely reduces the equipment uptime.

These issues above with the physical monitoring system highlight the critical imperative to develop a soft-sensor-based alternative.

### 2.4. Machine Learning Methods

This study evaluates a variety of machine learning models for the wire bow soft sensor, including SVR, MLP, DTR, RF, GB, and XGBoost. These models represent the core paradigms of kernel methods, tree-based ensembles, and neural networks, providing a multi-faceted validation of wire bow prediction. Among these, XGBoost is the primary focus of our discussion.

Given a dataset $D={\left\{\left(x_{i},y_{i}\right)\right\}}_{i=1}^{n}$. Here, $x_{i}\in R^{d}$ represents the *d*-dimensional feature vector containing process parameters (e.g., torques in N·m, feed speeds in mm/min) for the *i*-th sample, and $y_{i}\in R$ denotes the corresponding actual wire bow value (in mm). It is important to note that before model training, all input features and the target variable are normalized. Consequently, the internal mathematical derivations of these ML algorithms operate in a dimensionless functional space, and their internal parameters (e.g., weights, biases, and Lagrange multipliers) do not carry physical units. The main goal of these models is to learn a mapping function $f:R^{d}\to R$ so that the predicted wire bow value, represented as ${\hat{y}}_{i}=f\left(x_{i}\right)$ in this section, minimizes a specific loss function with respect to the actual value. The final output ${\hat{y}}_{i}$ is then inversely transformed to represent the wire bow prediction in its physical unit (mm).

#### 2.4.1. Support Vector Regression (SVR)

SVR [32,33] is a robust regression method grounded in statistical learning theory. It constructs a hyperplane to fit data within a predefined error tolerance, known as the ϵ-insensitive zone. Small residuals within this band are ignored, effectively enhancing model generalization. The objective of SVR is to minimize the following convex optimization problem:

(5) $$\min\frac{1}{2}{∥w∥}^{2}+C\sum_{i=1}^{n}\left(\xi_{i}+\xi_{i}^{*}\right)$$

where ${∥w∥}^{2}$ serves as the regularization term to prevent overfitting, and *C* is the penalty parameter that balances the complexity and the training error tolerance. The slack variables, $\xi_{i}$ and $\xi_{i}^{*}$, represent the upper and lower deviations of the training samples that fall outside the ϵ-insensitive zone. For the complex relationship between machine parameters and wire bow, the kernel trick (specifically the Radial Basis Function (RBF), denoted as $K(x_{i},x_{j})$) is utilized to map the input features into a high-dimensional space. The final prediction for a new input vector x is given by:

(6) $$\hat{y}=\sum_{i=1}^{n}\left(\alpha_{i}-\alpha_{i}^{*}\right)K\left(x_{i},x\right)+b$$

where $\alpha_{i}$ and $\alpha_{i}^{*}$ are the Lagrange multipliers obtained from the dual optimization problem, and *b* is the bias term.

#### 2.4.2. Multilayer Perceptron (MLP)

MLP [34] is a foundational feedforward artificial neural network that serves as a universal function approximator. It consists of an input layer, one or more hidden layers, and an output layer, with neurons between adjacent layers fully connected via weight matrices and bias vectors. For an MLP with a single hidden layer, the forward propagation process is formulated as:

(7) $$\hat{y}=f_{out}\left(W^{\left(2\right)}\cdot g\left(W^{\left(1\right)}x+b^{\left(1\right)}\right)+b^{\left(2\right)}\right)$$

where $W^{\left(1\right)},b^{\left(1\right)}$ and $W^{\left(2\right)},b^{\left(2\right)}$ represent the weights and biases for the hidden and output layers, respectively. g(·) denotes the non-linear activation function, such as the Rectified Linear Unit (ReLU), and $f_{out}\left(\cdot \right)$ is the output activation function. The model learns the optimal parameters θ={W,b} by minimizing the Mean Squared Error (MSE) through the backpropagation algorithm:

(8) $$\min_{\theta }\frac{1}{n}\sum_{i=1}^{n}{\left(y_{i}-{\hat{y}}_{i}\right)}^{2}$$

MLP excels at mapping the complex non-linear dynamics between sawing parameters and wire bow. However, it requires large-scale training data. Furthermore, performance depends heavily on careful hyperparameter tuning.

#### 2.4.3. Decision Tree Regression (DTR)

DTR [35] is a non-parametric method that recursively partitions the feature space into *M* disjoint regions, $R_{1},\ldots ,R_{M}$. In each terminal node (leaf), a constant value ${\hat{c}}_{m}$ is assigned, typically calculated as the mean of the training samples within that region (${\hat{c}}_{m}=\frac{1}{N_{m}}\sum_{x_{i}\in R_{m}}y_{i}$). The final prediction for a new input vector x is expressed as the sum of these regional constants weighted by an indicator function:

(9) $$\hat{y}=\sum_{m=1}^{M}{\hat{c}}_{m}I\left(x\in R_{m}\right)$$

where I(·) is the indicator function, which equals 1 if x falls into region $R_{m}$ and 0 otherwise. DTR offers computational efficiency and interpretability. However, it is sensitive to noise and prone to overfitting.

#### 2.4.4. Random Forest (RF)

RF [36] is an ensemble method based on Bootstrap Aggregating (Bagging). It enhances robustness by aggregating multiple de-correlated decision trees. The training generates *T* distinct subsets via bootstrap sampling. For each subset, a decision tree $h_{t}\left(x\right)$ is grown. Crucially, only a random subset of *k* features is considered at each node split. This strategy ensures diversity among trees. For the wire bow prediction problem, the final ensemble prediction $\hat{y}$ is the arithmetic mean of all individual tree outputs:

(10) $$\hat{y}=\frac{1}{T}\sum_{t=1}^{T}h_{t}\left(x\right)$$

where $h_{t}\left(x\right)$ represents the prediction from the *t*-th decision tree. This mechanism of random feature selection and sample re-sampling significantly reduces the model’s variance. Consequently, RF is resilient to noise and less prone to overfitting than single decision trees.

#### 2.4.5. Gradient Boosting (GB)

GB [37] is a sequential ensemble method. It constructs a robust model by incrementally adding weak learners, typically shallow decision trees. Each subsequent learner is designed to fit the negative gradient of the loss function with respect to the current ensemble’s predictions. Using a forward stage-wise additive strategy, the model F(x) updates at the *m*-th iteration:

(11) $$F_{m}\left(x\right)=F_{m-1}\left(x\right)+\nu \cdot h_{m}\left(x\right)$$

where $F_{m-1}\left(x\right)$ represents the model accumulated from previous steps, ν is the learning rate (shrinkage factor) utilized to scale the contribution of each tree and mitigate overfitting. $h_{m}\left(x\right)$ is the base learner at step *m*. The objective of $h_{m}\left(x\right)$ is to fit the *pseudo*-residuals $r_{im}$, which are derived from the differentiable loss function $L(y_{i},F\left(x_{i}\right))$:

(12) $$r_{im}=-{\left.\frac{\partial L\left(y_{i},F\left(x_{i}\right)\right)}{\partial F(x_{i})}\right|}_{F\left(x_{i}\right)=F_{m-1}\left(x_{i}\right)},\;i=1,\ldots ,n.$$

After *M* iterations, the final prediction $\hat{y}$ is obtained by aggregating the initial guess and all subsequent updates:

(13) $$\hat{y}=F_{M}\left(x\right)=F_{0}\left(x\right)+\sum_{m=1}^{M}\nu \cdot h_{m}\left(x\right)$$

where $F_{0}\left(x\right)$ is the initial constant prediction. By iteratively addressing the residual errors in the functional space, GB achieves high precision in capturing complex input-output relationships, though it requires meticulous regularization to maintain generalization capability.

#### 2.4.6. eXtreme Gradient Boosting (XGBoost)

XGBoost [38] is a highly efficient implementation of Gradient Boosting. It achieves superior predictive performance and computational speed. The algorithm employs an additive training strategy. The *i*-th prediction, ${\hat{y}}_{i}$, aggregates outputs from *K* base learners (Classification and Regression Trees (CART)):

(14) $${\hat{y}}_{i}=\sum_{k=1}^{K}f_{k}\left(x_{i}\right),\;f_{k}\in F$$

where F denotes the functional space of all possible CART models, and $x_{i}$ represents the input feature vector. To balance accuracy and model complexity, the objective function L includes a loss function *L* and a structural regularization term Ω:

(15) $$L=\sum_{i=1}^{n}L\left(y_{i},{\hat{y}}_{i}\right)+\sum_{k=1}^{K}\Omega \left(f_{k}\right)$$

where $y_{i}$ is the actual target value. The complexity of a single tree $f_{k}$ is defined as $\Omega \left(f_{k}\right)=\gamma N+\frac{1}{2}\lambda \sum_{j=1}^{N}w_{j}^{2}$. Here, *N* denotes the number of leaf nodes, $w_{j}$ represents the weight of the *j*-th leaf, and γ and λ are regularization coefficients. To optimize the objective at the *t*-th iteration, a second-order Taylor expansion is applied to approximate the loss:

(16) $$L^{\left(t\right)}≃\sum_{i=1}^{n}\left[g_{i}f_{t}\left(x_{i}\right)+\frac{1}{2}h_{i}f_{t}^{2}\left(x_{i}\right)\right]+\Omega \left(f_{t}\right)$$

where $g_{i}=\partial_{{\hat{y}}_{i}^{\left(t-1\right)}}L\left(y_{i},{\hat{y}}_{i}^{\left(t-1\right)}\right)$ and $h_{i}=\partial_{{\hat{y}}_{i}^{\left(t-1\right)}}^{2}L\left(y_{i},{\hat{y}}_{i}^{\left(t-1\right)}\right)$ denote the first and second-order derivatives of the loss function. By minimizing this approximate objective, the optimal structure and leaf weights of the *t*-th tree $f_{t}$ are obtained. The final prediction for a new input vector x is given by:

(17) $$\hat{y}=\sum_{k=1}^{K}f_{k}\left(x\right)$$

In the MWS process, the formation of wire bow is a complex non-linear response driven by the multi-system coupling of the spindle, wire web, and the feeding mechanism. XGBoost is particularly suitable for this task as it can automatically capture the complex interactions among process parameters (e.g., feed rate, wire speed, and slurry flow) that traditional linear models may overlook.

The core mathematical formulations governing these six ML models are consolidated in Table 1.

### 2.5. Two-Stage Hyperparameter Optimization

The preceding analysis of machine learning models highlights that hyperparameter tuning is critical to predictive accuracy and overfitting control. Given the hyperparameter space Θ, which comprises discrete parameters (e.g., tree depth and number of neurons) and continuous parameters (e.g., learning rate and regularization coefficients), a two-stage hybrid optimization strategy is proposed.

The hyperparameter space is defined as $\Theta =\Theta_{d}\times \Theta_{c}$, where $\Theta_{d}$ is the discrete subspace and $\Theta_{c}$ is the continuous subspace. The objective function f(θ) is formulated as follows:

(18) $$f\left(\theta \right)=L_{cv}$$

where $M_{\theta }$ denotes the model trained with hyperparameter vector θ, $L_{cv}$ is the *k*-fold cross-validation loss.

**Stage I: Discrete Grid Search.** In the first stage, a global coarse search is performed over the discrete subspace $\Theta_{d}$ using an exhaustive grid search:

(19) $$\theta_{d}^{*}=\arg\min_{\theta_{d}\in \Theta_{d}}f\left(\theta_{d},\theta_{c}^{ini}\right)$$

where $\theta_{c}^{ini}$ is the preset baseline vector for continuous parameters (typically the median of their respective ranges).

**Stage II: Local Bayesian Optimization.** Given the optimal discrete parameters $\theta_{d}^{*}$ identified in Stage I, the second stage conducts a fine-grained search over the continuous hyperparameters. The optimization is restricted to the subspace $\Theta_{\mathrm{local}}=\left\{\theta_{d}^{*}\right\}\times \Theta_{c}$. A Bayesian Optimization (BO) [39] framework utilizing a Gaussian Process (GP) surrogate model is employed. The Matérn 5/2 kernel is adopted to capture the spatial correlation:

(20) $$k\left(\theta_{c},\theta_{c}^{\prime }\right)=\sigma_{f}^{2}\left(1+\frac{\sqrt{5}r}{l}+\frac{5r^{2}}{3l^{2}}\right)\exp\left(-\frac{\sqrt{5}r}{l}\right),\;r=∥\theta_{c}-\theta_{c}^{\prime }∥$$

where *l* is the length scale and $\sigma_{f}^{2}$ is the signal variance. To leverage the information from Stage I, an improved Expected Improvement (EI) acquisition function is introduced:

(21) $$\alpha_{EI}\left(\theta_{c}\right)=E\left[\max\left(f_{grid}^{*}-f\left(\theta_{d}^{*},\theta_{c}\right),0\right)\right]\cdot w\left(\theta_{c}\right),\;f_{grid}^{*}=f\left(\theta_{d}^{*},\theta_{c}^{ini}\right)$$

Here, $w\left(\theta_{c}\right)=\exp\left(-\frac{∥\theta_{c}-\theta_{c}^{ini}{∥}^{2}}{2\sigma^{2}}\right)$ is a distance-based weighting term that guides the search to prioritize regions around the validated baseline. The optimal continuous parameters are updated iteratively for *T* rounds:

(22) $$\theta_{c}^{\left(t+1\right)}=\arg\max_{\theta_{c}\in \Theta_{c}}\alpha_{EI}^{\left(t\right)}\left(\theta_{c}\right),\;t=1,\ldots ,T$$

### 2.6. Establishment of the Wire Bow Soft Sensor

**Figure 5:**
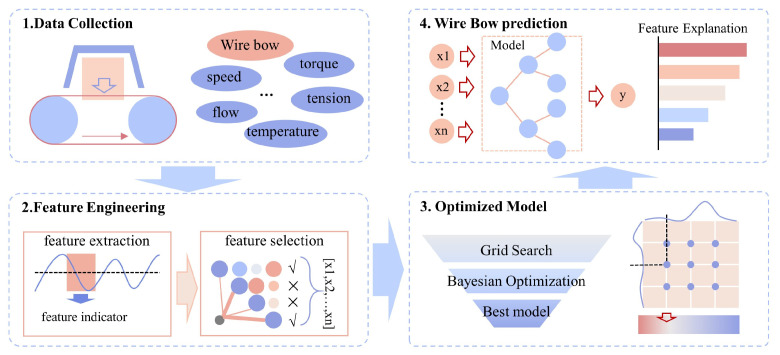
Framework of the wire bow soft sensor.

Figure 5 illustrates the systematic modeling workflow for the wire bow soft sensor. Based on the acquired sawing process parameters and measured wire bow data, an initial feature set is first constructed through steady-state filtering and feature extraction. Subsequently, feature selection and dimensionality reduction are performed using correlation analysis and collinearity diagnostics to refine the input space. On this basis, a hybrid hyperparameter optimization strategy, coupling grid search with Bayesian optimization, is employed to train the regression models. The training and validation phases are executed via a *k*-fold cross-validation framework. Finally, the generalization performance of the models is evaluated using an independent test set to achieve a precise prediction of the wire bow.

**Figure 6:**
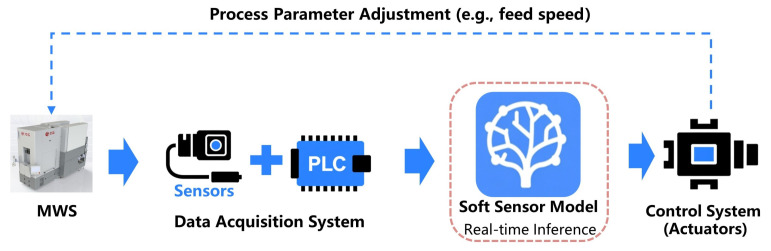
The deployment architecture for real-time wire bow prediction and monitoring.

To bridge the gap between model development and industrial application, Figure 6 illustrates the deployment architecture for the practical realization of this framework in a plant setting. The deployment workflow involves four main stages: (1) Real-time Data Acquisition, where process parameters are streamed from the sensors and PLC via industrial protocols (e.g., OPC UA); (2) Online Inference, where the trained soft sensor model instantly provides a real-time estimation of the wire bow; (3) Monitoring & Decision Support, where the predicted value is monitored to alert operators of abnormal bowing; and (4) Closed-Loop Feedback, where the control system (actuators) executes dynamic process parameter adjustments (e.g., modifying the feed speed). While this study primarily validates the high-precision prediction for the wire bow required for the first three stages, the proposed architecture fully integrates the fourth stage, serving as a critical framework for future automated process modifications.

## 3. Experiments and Result Analysis

This section presents the experimental details and analysis results. First, the data acquisition setup and feature selection process are described. Next, the prediction model using XGBoost is constructed and optimized. Its performance is then evaluated against other algorithms. Furthermore, the model is tested across different spatial positions of the wire web. Finally, the key influencing factors are interpreted using SHAP analysis.

### 3.1. Data Acquisition and Preprocessing

**Figure 7:**
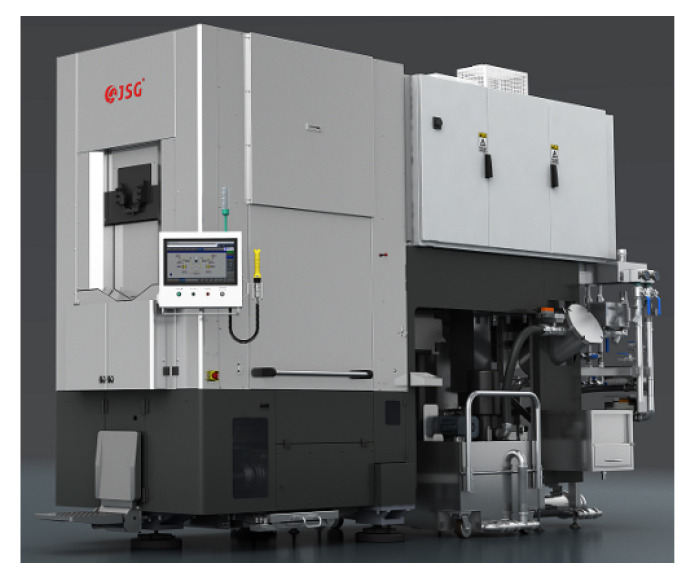
Diamond multi-wire sawing machine.

**Figure 8:**
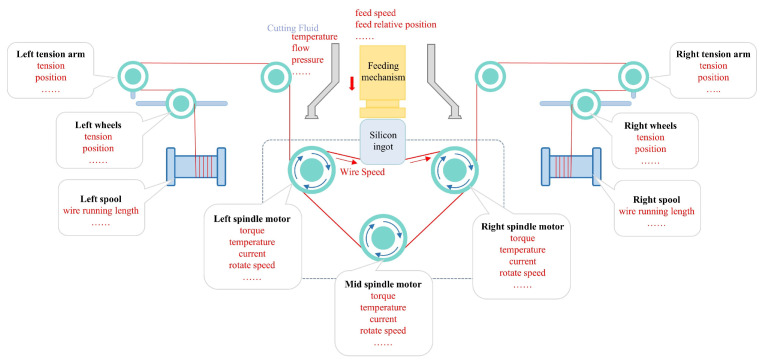
Diagram of the data acquisition points in MWS machine.

The experimental setup utilized the diamond MWS machine WS950XH manufactured by Zhejiang Jingsheng Mechanical & Electrical Co., Ltd., Shaoxing, China, as shown in Figure 7. The dataset comprises data from 12 complete production runs collected during actual cutting operations in March 2025. Notably, all 12 runs are conducted using the same silicon ingot type and the same diamond wire batch under an identical machine configuration. Specifically, the wire bow data are obtained using the monitoring system described in Section 2.2. There are approximately 100 wire bow measurements in each run. To ensure data accuracy, the monitoring system underwent re-calibration and lens cleaning after each cutting cycle. Meanwhile, process data are acquired directly via the Programmable Logic Controller (PLC) of Shenzhen Inovance Technology Co., Ltd. from Shenzhen, China, covering key subsystems (e.g., the main spindle and the cutting fluid system). A total of 103 distinct process features are acquired. The schematic diagram of the typical data acquisition points is illustrated in Figure 8.

**Figure 9:**
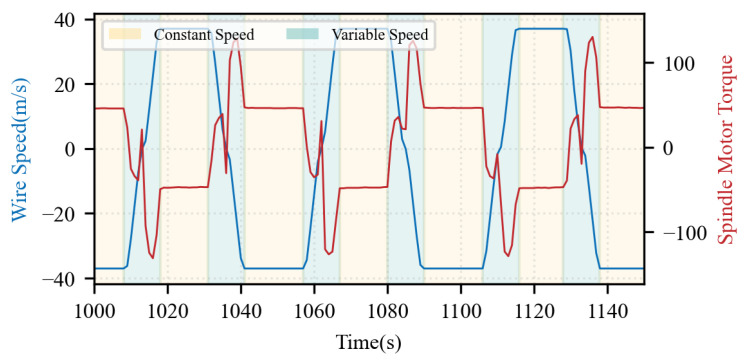
Wire speed and spindle motor torque fluctuations across different motion phases.

This study focuses on the steady-state operating conditions of the wire sawing machine. Figure 9 shows the wire speed and spindle motor torque fluctuations across different motion phases during a specific time period of the wire sawing process. The operating conditions exhibit significant cyclical characteristics due to the periodic starting, stopping, reversing, and speed regulation of the spindle motor. Specifically, each cycle can be categorized into three distinct phases: acceleration, constant-speed (steady-state), and deceleration. In this study, constant-velocity subsets are segmented from the raw time-series data for each cutting cycle. For each extracted subsequence, the mean values of the process monitoring parameters are computed and paired with the corresponding averaged steady-state wire bow measurements to construct sample pairs (x,y). These pairs serve as the input feature vectors and target labels for the subsequent modeling phase.

Prior to feature selection and model training, rigorous data preprocessing is conducted to ensure data quality and eliminate dimensional biases. First, to handle occasional missing values caused by PLC communication delays, a linear interpolation method is applied to estimate isolated missing points, ensuring the temporal continuity of the physical signals. Subsequently, all process variables are subjected to Min-Max normalization, scaling them to a unified dimensionless range of [0, 1]. This normalization step is executed before feature selection to ensure that the subsequent variance thresholding evaluates the true relative fluctuations of the signals, preventing variables with large physical absolute values from dominating the selection process.

### 3.2. Feature Selection

The MWS process is inherently complex, involving a high-dimensional set of online monitoring variables across multiple subsystems. Directly utilizing all raw variables as model inputs would introduce significant noise and irrelevant features, leading to computational inefficiency. Besides, the “curse of dimensionality” due to feature redundancy and multicollinearity can impair the model’s generalization ability and interpretability. To address this issue, the features are selected as follows:

Monitoring parameters lacking direct physical correlation to wire bow formation are excluded firstly. This selection is guided by practical production experience and process mechanism analysis. Specifically, unrelated variables, such as bearing housing temperature and traversing motor current, are removed. Consequently, irrelevant dimensions of the feature space are eliminated.

**Figure 10:**
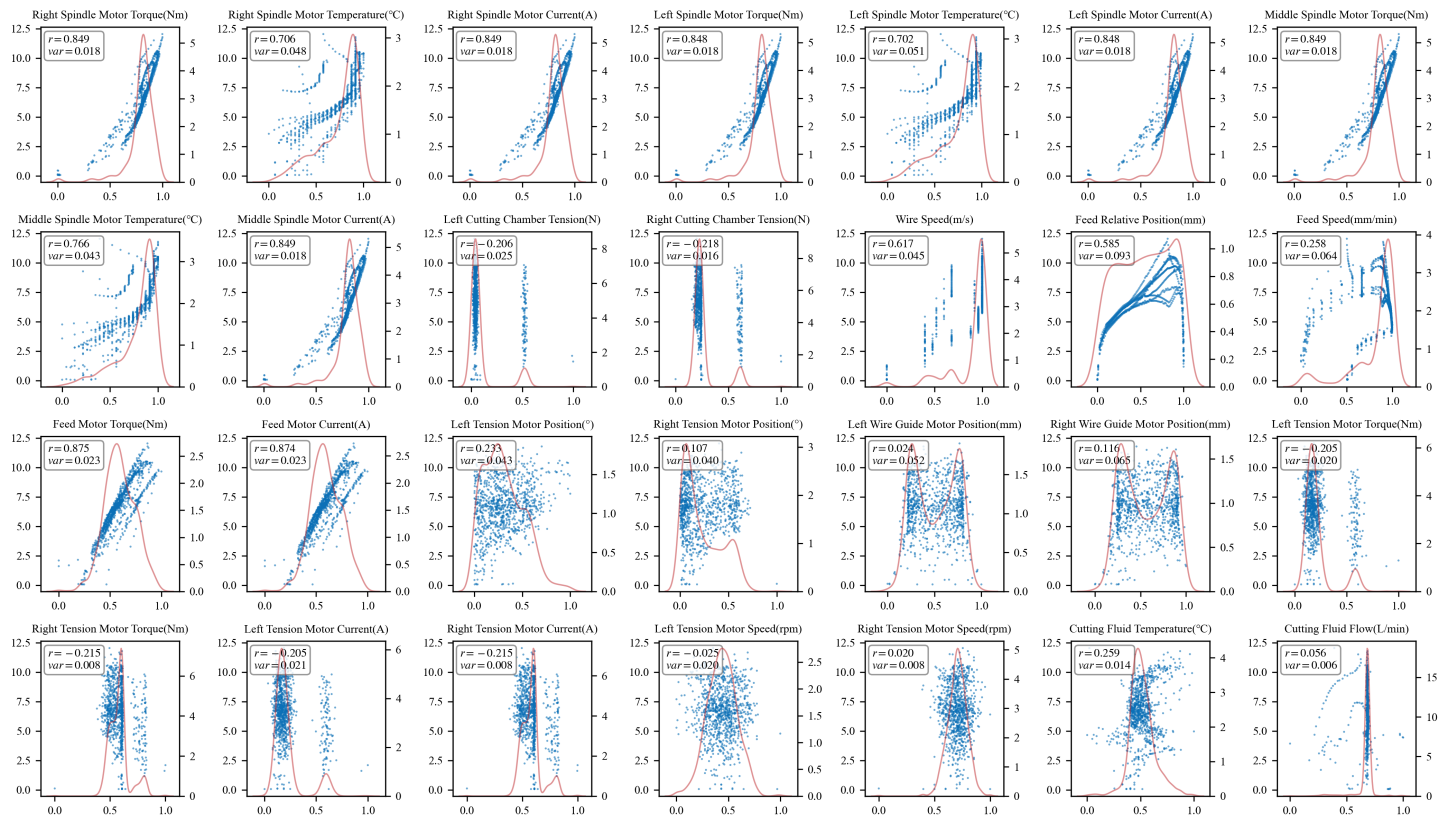
Correlation and variance analysis of features. In each subplot, the horizontal axis represents the normalized value of an individual feature, and the vertical axis denotes the wire bow measurement. The red curve illustrates the probability density distribution of the feature.

**Figure 11:**
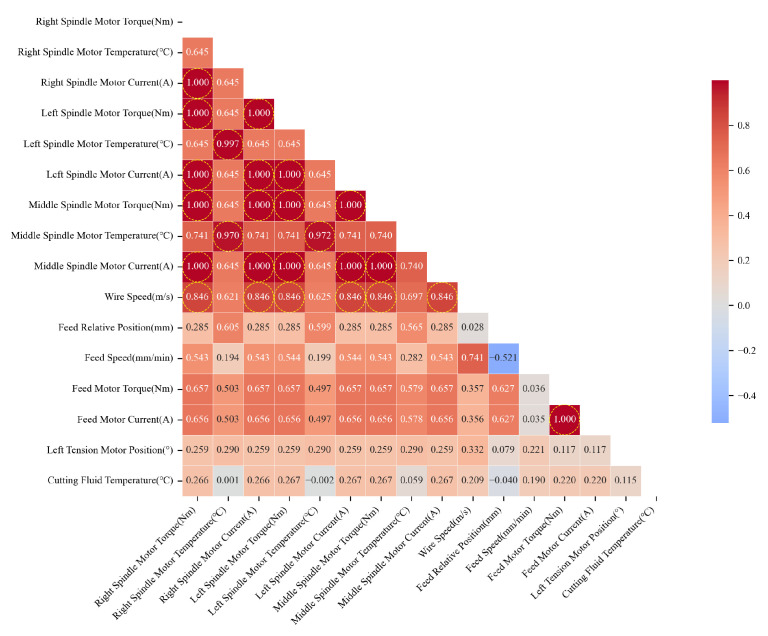
The correlation heatmap between the variables.

Then, a statistical selection strategy combining variance thresholding and correlation analysis is further employed for the retained features as illustrated in Figure 10. This strategy serves as a preliminary filter to reduce the dimensionality of the raw feature space by removing near-constant variables and those with negligible linear associations. The features are ranked by variance, and the top 20 variables exhibiting the highest variance are retained empirically, as variables with lower variance typically contain limited discriminative information. Subsequently, correlation analysis is performed. Features exhibiting at least a ’moderate’ correlation (Pearson coefficient |r|≥0.3) with the target wire bow *y* are retained. According to widely accepted statistical criteria for exploratory data analysis [40], |r|≥0.3 represents a medium effect size, below which variables are generally considered to have limited practical significance. Finally, multicollinearity among coupled parameters is addressed. Features with high mutual correlation (Pearson coefficient |r|>0.8) are grouped. The correlation heatmap is presented in Figure 11. Within each group, only the representative feature with the highest correlation to *y* is preserved. For instance, high multicollinearity is observed among the current, torque, and temperature of the spindle motors. This phenomenon is attributed to physical mechanism coupling. Only the spindle torque (r=0.845) is selected as the representative feature. Specifically, although the right and left spindle torques are highly correlated, practical experience indicates that their asymmetry is a key determinant of the wire bow. Therefore, both variables are retained.

Consequently, an optimized feature set is derived, as listed in Table 2. The selected features are target-correlated and mutually independent. Thus, a rigorous basis is established for reliable model development.

### 3.3. Model Construction

A wire bow prediction model is constructed using the XGBoost algorithm as a representative example. The construction and parameter optimization processes for other models follow an identical methodology.

#### 3.3.1. Dataset Partitioning

The dataset is constructed from 12 wire sawing runs. A chronological partitioning strategy is adopted to ensure temporal validity. Data from the first 10 runs (996 samples) constitute the training set. Conversely, data from the final 2 runs (211 samples) serve as the independent test set. This scheme simulates real-world production scenarios. Historical data is utilized to predict future conditions. Consequently, the model’s predictive performance is evaluated effectively.

#### 3.3.2. Hyperparameter Optimization Framework and Model Training

To determine the optimal hyperparameters and mitigate overfitting, a rigorous optimization framework is established based on 5-fold cross-validation. This process is conducted exclusively within the training set (996 samples). The implementation steps are as follows:

- **Data randomization and subset partitioning:** The 996 training samples are randomly shuffled. This random shuffling strategy is chosen for the hyperparameter optimization phase. It effectively breaks the strong temporal autocorrelation within individual production runs, providing a more uniform distribution for stable model learning and parameter convergence. Then, the shuffled data is uniformly divided into 5 mutually exclusive folds. Each fold contains approximately 199 samples. It is crucial to note that this process strictly excludes the independent production runs reserved for the final across-run testing, thereby preventing any data leakage.
- **Iterative validation:** Five rounds of training are conducted. In each round, one fold is selected as the validation set and the remaining four folds function as the training set. The validation set is excluded from parameter updates. Instead, it monitors loss function convergence and triggers early stopping to prevent overfitting.
- **Hyperparameter optimization:** The hybrid strategy combining grid search (Stage I) and Bayesian optimization (Stage II) is employed, as elaborated in Section 2.5. The objective function for optimization is the average mean absolute error (MAE) derived from the 5-fold cross-validation, with the Bayesian optimization configured to run for 1000 iterations.

**Figure 12:**
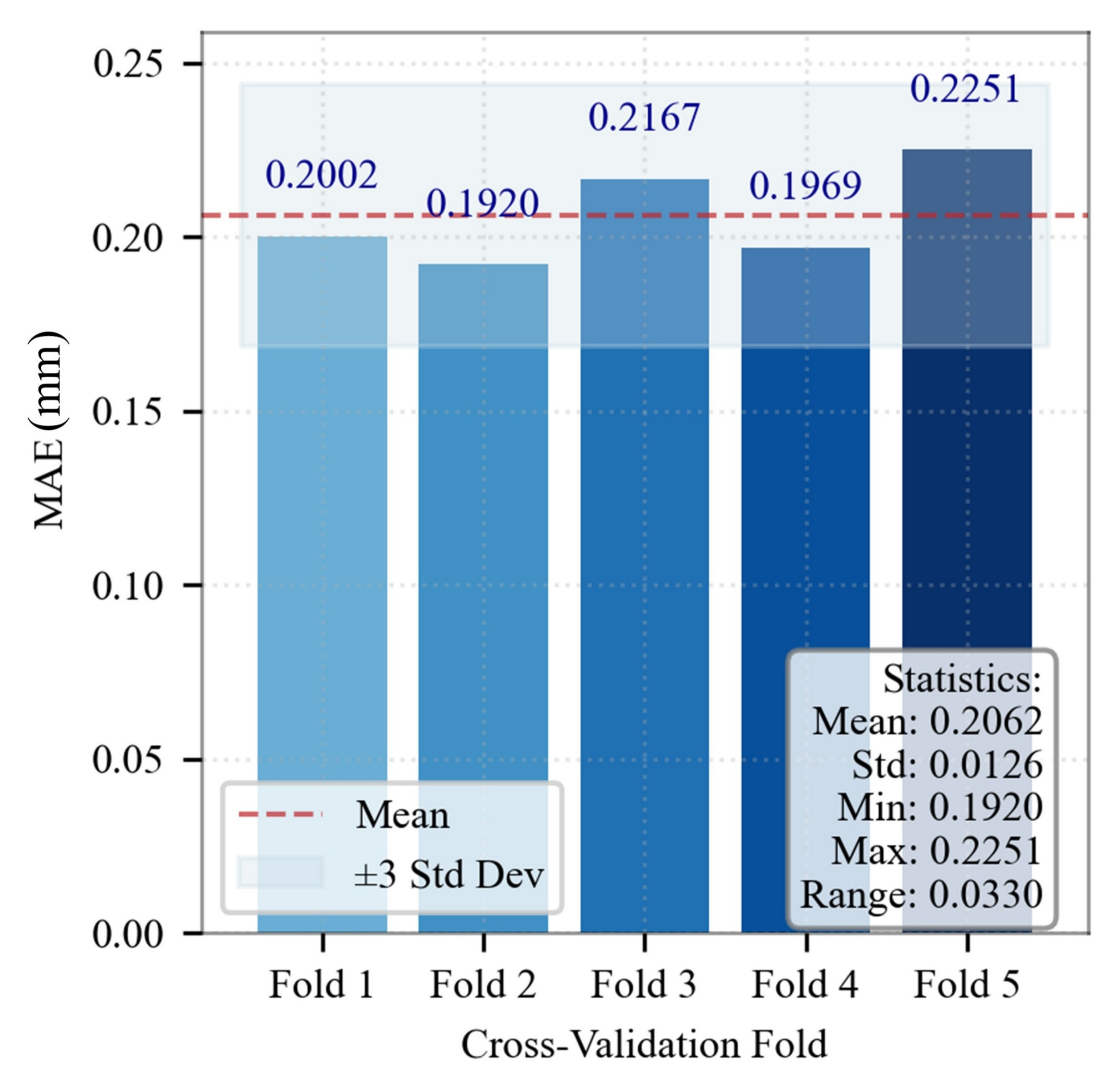
MAE on each validation fold for the optimal hyperparameters.

The specific hyperparameter search spaces and the optimal values are detailed in Table 3. The MAE on each validation fold for the optimal hyperparameter combination are presented in Figure 12. The average MAE is 0.2062 ± 0.0126 mm, which is significantly lower than the engineering tolerance threshold (0.5 mm). Low fluctuation in prediction errors is observed across folds. Consequently, stable predictive performance is demonstrated.

Ultimately, based on the optimal hyperparameter combination identified from the validation phase, the model is retrained using the entire training dataset (all 996 samples) for the final assessment on the independent test set.

#### 3.3.3. Model Testing

The performance of the models is evaluated using the Mean Absolute Error (MAE), Mean Squared Error (MSE), Root Mean Squared Error (RMSE), and the Coefficient of Determination ($R^{2}$):

(23) $$\begin{array}{cc} \mathrm{MAE} & =\frac{1}{n}\sum_{i=1}^{n}\left|y_{i}-{\hat{y}}_{i}\right| \end{array}$$

(24) $$\begin{array}{cc} \mathrm{MSE} & =\frac{1}{n}\sum_{i=1}^{n}{\left(y_{i}-{\hat{y}}_{i}\right)}^{2} \end{array}$$

(25) $$\begin{array}{cc} R^{2} & =1-\frac{\sum_{i=1}^{n}{\left(y_{i}-{\hat{y}}_{i}\right)}^{2}}{\sum_{i=1}^{n}{\left(y_{i}-\bar{y}\right)}^{2}} \end{array}$$

(26) $$\begin{array}{cc} \mathrm{RMSE} & =\sqrt{\frac{1}{n}\sum_{i=1}^{n}{\left(y_{i}-{\hat{y}}_{i}\right)}^{2}} \end{array}$$

where *n* is the number of samples, $y_{i}$ denotes the actual value, ${\hat{y}}_{i}$ represents the predicted value, and $\bar{y}$ is the mean of the actual values.

**Figure 13:**
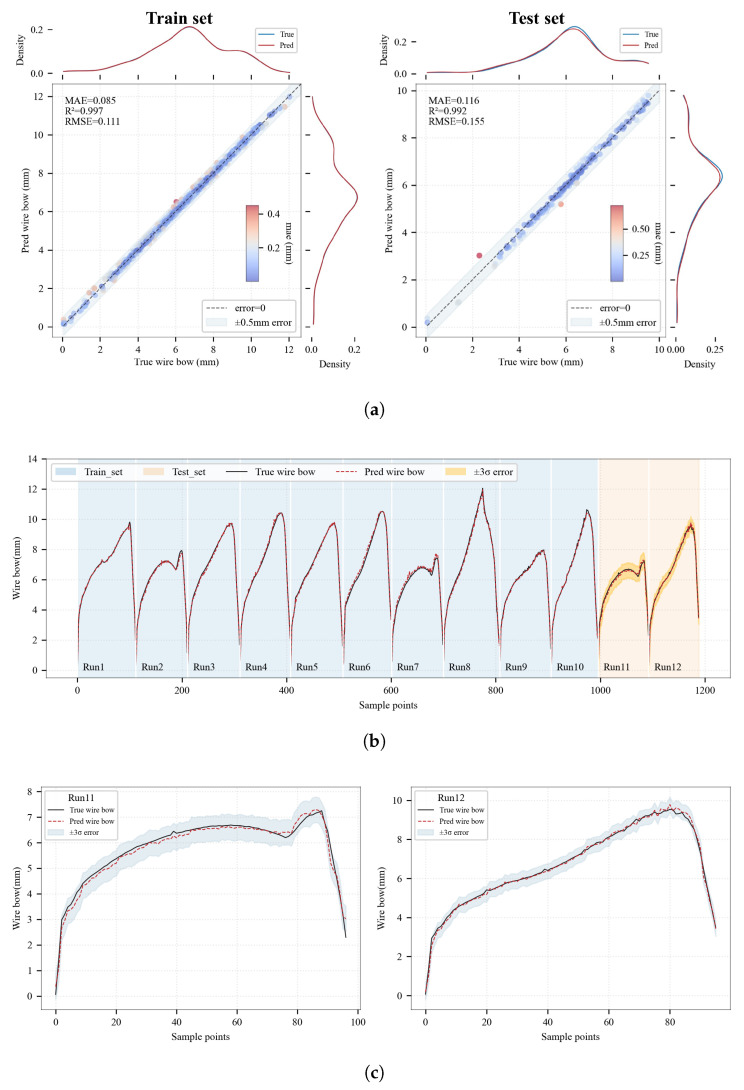
Comprehensive evaluation of the wire bow prediction model. (**a**) Joint distribution plot comparing the actual measured wire bow with the predicted values. The main scatter plot illustrates the point-wise absolute errors, with the central dashed line representing perfect estimation and the shaded region indicating the acceptable ±0.5 mm engineering tolerance band. The marginal plots show the probability density distributions of the measured and estimated values, respectively. (**b**) Time-series observation of the wire bow predictions across different production runs. The light blue shaded region (Runs 1–10) indicates the data used for model training, whereas the light orange shaded region (Runs 11–12) represents the completely independent across-run test set. (**c**) Prediction results for Run 11 and Run 12. The x-axis denotes the sampling sequence as the cutting proceeds.

The wire bow prediction is performed on the two runs within the test set, with the results illustrated in Figure 13. Specifically, Figure 13a illustrates a comparative analysis of the model’s performance on both the training and testing sets. For the training data, the R^2^ reaches 0.997 with an MAE of 0.085 mm. On the testing data, the *R*^2^ remains as high as 0.992, while the MAE and RMSE are 0.116 mm and 0.155 mm, respectively. These metrics demonstrate minimal prediction error and high stability. Regarding engineering tolerance, 98.96% of the prediction deviations fall within the 0.5 mm specification range, fully satisfying the precision requirements for actual production control. As observed in the Probability Density Function (PDF) distribution of predicted versus actual values in Figure 13a, the two curves overlap significantly. This indicates that the model accurately captures the data distribution characteristics.

Further analyzing from a time-series perspective, the comparison between predicted trends and actual measurements for the test set (Run 11 and Run 12, corresponding to Figure 13c) is illustrated. It can be observed that the wire bow gradually increases as the cutting proceeds. Notably, Run 2, 7 and 11 exhibit a significantly lower overall wire bow compared to other runs. This technological difference stems from intentional variations in the machining process parameters. Specifically, these specific runs are operated with adjusted feed speeds and different wire speeds during the steady-state cutting period, which directly contributed to the reduction in wire bow. After reaching a certain magnitude, it decreases rapidly until the cutting process is completed. The results show that the predicted curves align closely with the actual curves in the temporal dimension, with no observable lag or distortion. It demonstrates strong generalization capability and temporal consistency, making it effectively applicable to real-world monitoring scenarios across different wire saw runs.

### 3.4. Comparative Analysis of Various Model Performance

Six machine learning models are systematically evaluated for wire bow prediction. The candidates include SVR, DTR, RF, GB, MLP, and XGBoost. To ensure a fair and rigorous comparison, the core hyperparameters of all baseline models undergo systematic optimization to maximize their respective predictive capabilities. For instance, the key parameters for tree-based ensembles, such as the number of estimators in the RF model, are carefully optimized. Detailed optimized hyperparameters for these comparison models are listed in Table 4. This assessment focuses on prediction accuracy and engineering applicability.

**Figure 14:**
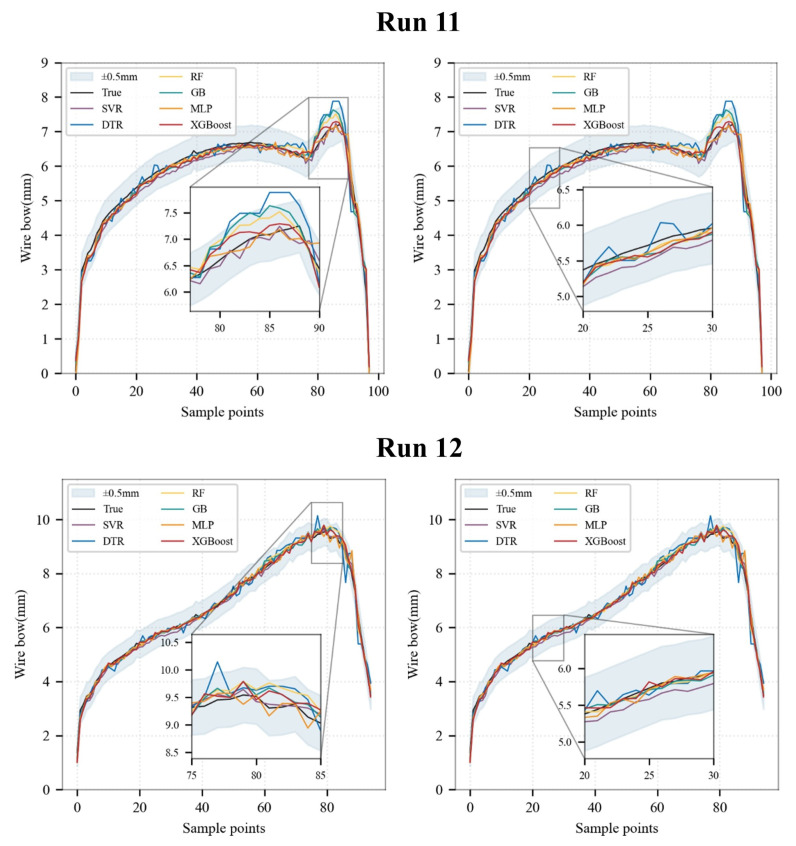
Comparison of the wire bow prediction trajectories among different machine learning models for the independent test runs (Run 11 and Run 12).

As illustrated in the fitting trend analysis of wire bow evolution during the cutting process Figure 14, all models effectively capture the overall upward trend of wire bow values as the cutting process proceeds. Furthermore, during the phase of rapid wire bow decline at the end of the whole cutting cycle, the prediction curves of all models track the actual variation trends reasonably well.

However, significant discrepancies are observed in local fitting accuracy and stability among the models. Specifically, the DTR model exhibits the most pronounced fluctuations, with substantial deviations evident in both the stable ascending stage and regions of abrupt wire bow changes. In contrast, the remaining models demonstrate superior fitting smoothness during the steady ascending phase, maintaining a close alignment with the ground truth curve.

Nevertheless, at critical transition stages characterized by significant wire bow inflections shown in Figure 14, the DTR, RF, GB, and XGBoost models all exhibit a certain degree of positive bias. Conversely, the SVR and MLP models maintain minor fluctuations around the actual values, demonstrating superior operational stability under these working conditions.

**Figure 15:**
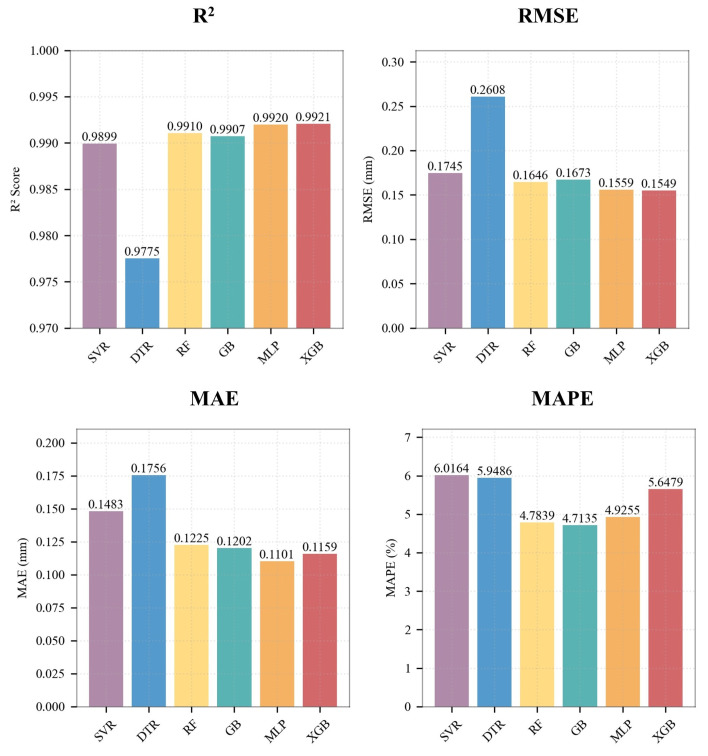
Comparison of the model prediction performance metrics.

Based on the analysis of quantitative evaluation metrics (Figure 15), the XGBoost model outperforms all others in terms of $R^{2}$, RMSE, and MAE, demonstrating the highest goodness of fit and the lowest prediction error. In contrast, the DTR model exhibits the largest errors across these metrics. Notably, regarding the mean absolute percentage error (MAPE) metric, the MLP model achieved the lowest value, indicating a specific advantage in relative error control.

### 3.5. Wire Bow Prediction in Various Positions

**Figure 16:**
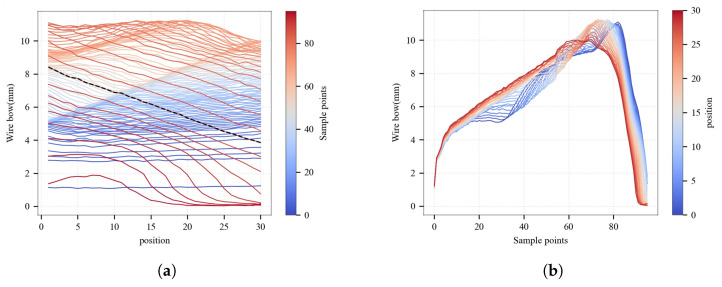
Spatiotemporal variations of the wire bow during the multi-wire sawing process. (**a**) Wire bow profiles across different axial positions at various sampling instants. The color gradient from blue to red indicates the progression of the cutting process from the initial to the final stages. (**b**) Temporal evolution trends of the wire bow at specific axial positions throughout the cutting process. The color mapping from blue to red denotes the spatial distribution of the axial positions, ranging from position 1 to position 30.

During the MWS process, wire bow exhibits variations along the axial direction of the spindle, as demonstrated in Figure 3. This phenomenon is due to non-uniform tension distribution, diamond wire wear, uneven cutting fluid flow fields, and other factors. The entire wire web is divided into 30 zones. Position 1 represents the wire-in side, and position 30 represents the opposite side. As illustrated in Figure 16a, the measured wire bow profile (indicated by the black dashed line) reveals that the variation in wire bow values across the entire web can reach 4 mm. This highlights the spatial heterogeneity of local force states within the cutting zone. Furthermore, the temporal evolution patterns of the wire bow at different axial positions exhibit significant disparities (Figure 16a), suggesting that its dynamic response characteristics are position-dependent. Consequently, it is essential to analyze and predict the wire bow across different positions.

Three representative zones are selected for predictive modeling of wire bow: the head zone (near the wire-in side, position = 1), the tail zone (near the wire-out side, position = 30), and the middle zone (located in the center of the wire web, position = 15). Based on the comparative analysis in Section 3.4, the XGBoost algorithm, which demonstrated the superior performance in terms of accuracy and generalizability, is employed for modeling in these specific positions. The model construction and training procedures are consistent with the mean wire bow prediction model above. The hyperparameter optimization results are listed in Table 5, and the testing results are illustrated in Figure 16.

**Figure 17:**
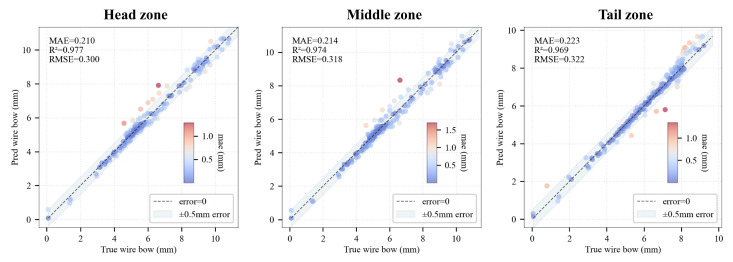
Evaluation of the wire bow estimation model across different spatial zones (Head, Middle, and Tail) of the wire web.

Figure 17 illustrate the performance of the wire bow prediction models developed for the head, middle, and tail zones, respectively. Compared to the global mean wire bow prediction model, the prediction accuracy of the regional models shows a slight decline, with the coefficient of determination ($R^{2}$) decreasing to approximately 0.97. Specifically, the models for the head and central zones exhibit notable prediction deviations in the medium wire bow range (nearly 5–7 mm), and the tail zone model demonstrates relatively limited predictive capability for larger wire bow values (greater than 8 mm).

The reduction in accuracy is attributed to the nature of the process data. Parameters collected by current equipment (e.g., the spindle torque) are axially integrated quantities. These aggregate metrics fail to fully capture the pressure gradients and local mechanical states distributed along the spindle. Consequently, there are still systemic limitations when characterizing local spatial details. Nevertheless, the MAE of each regional model remains within an industrially acceptable range (0.5 mm), indicating that this modeling approach possesses practical applicability.

### 3.6. Feature Interpretation

**Figure 18:**
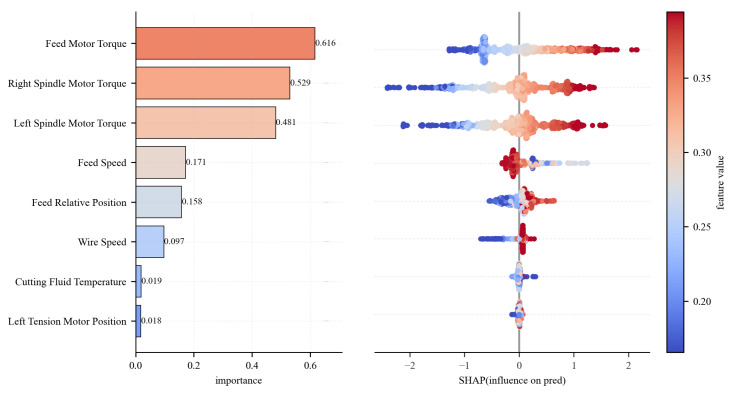
SHAP beeswarm plot.

**Figure 19:**
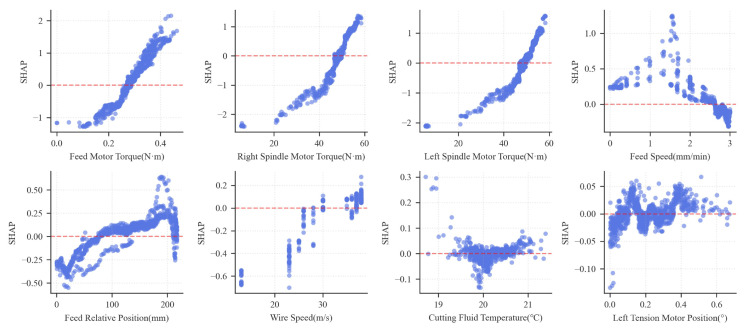
SHAP dependency plot.

To further elucidate the relationship between features and wire bow, this section adopts the SHAP (SHapley Additive exPlanations) method [41] to systematically quantify the contribution of each feature to the model’s predictions and analyze their modes of action. The magnitude of the SHAP values reflects the importance of each feature, while the positive or negative signs indicate whether a feature promotes or inhibits the prediction results. Figure 18 shows the SHAP beeswarm plot, and Figure 19 indicates the SHAP dependency plot. These two plots reveal the impact of key features on wire bow prediction.

The results show that the feed motor torque exerts the most significant influence on the wire bow prediction model, exhibiting a SHAP contribution value of 0.616. This is followed by the left and right spindle torques, with contribution values of 0.529 and 0.481, respectively. The SHAP dependency plot further reveals that both the feed motor torque and spindle torques show a significant positive correlation with the wire bow. Specifically, as these torque values increase, the predicted wire bow tends to rise.

From the perspective of cutting mechanics, these torques collectively reflect the load characteristics of the sawing process. The feed torque primarily originates from the resistance encountered when pressing the ingot down against the wire web, while the spindle torque corresponds to the contact resistance at the interface between the wire web and the ingot. Fundamentally, both torque categories represent the interaction forces between the wire saw and the ingot. Therefore, they exhibit a strong coupling relationship with the wire bow, which is the characteristic physical quantity representing the cutting state.

In contrast, process parameters such as wire speed, feed position, and feed speed exert a relatively limited influence. These parameters primarily affect wire bow formation indirectly by modulating the cutting process conditions. Meanwhile, according to the SHAP dependence plots, the feed speed exhibits a distinct non-monotonic trend: SHAP values peak within the 1.5–2.0 mm/min interval and subsequently decline as speed increases further, suggesting an optimal process window beyond which the influence of feed speed diminishes. The wire speed demonstrates a threshold effect, where SHAP values transition from a near-zero baseline to a sharp positive surge at approximately 30 m/s, indicating a shift in the material removal mechanism at this critical point. The impact of the relative feed position is stage-dependent. It shows a positive correlation in the initial stage but exhibits significant fluctuations after the stroke exceeds 150 mm, likely reflecting the cumulative wear of the diamond wire during prolonged cutting processes.

**Figure 20:**
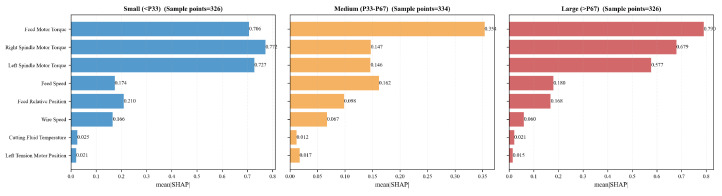
Comparison of feature contributions under various wire bow scales using the mean SHAP.

Furthermore, subgroup analysis based on wire bow reveals that motor torques maintain high importance across various wire bow scales, as shown in Figure 20. Specifically, the influence of the feed motor torque is particularly pronounced under medium (P33–P67, representing the 33rd to 67th percentile range) and large wire bow conditions (>P67). Conversely, under small wire bow conditions (<P33), the right spindle torque emerges as the dominant influencing factor.

The result further corroborates the practical significance of motor torque as a critical engineering indicator reflecting process stability. Simultaneously, they indicate that the key feature parameters requiring prioritized regulation and monitoring vary according to the specific stage of the wire bow.

## 4. Conclusions and Future Work

The critical challenge of real-time wire bow monitoring in harsh diamond MWS environments is addressed. A high-precision machine learning-based soft sensor is developed. Unlike traditional mechanistic models that rely on idealized assumptions or physical sensors that suffer from contamination and spatial constraints, the proposed data-driven approach establishes a reliable mapping between readily measurable process parameters and the wire bow.

First, through statistical screening and correlation analysis, variables with high physical relevance are identified as the primary indicators of wire bow, a dimensionality reduction that significantly enhances computational efficiency. Secondly, among the six comparative algorithms, the XGBoost model with two-stage hyperparameter optimization exhibits the best performance on the independent test set, achieving an $R^{2}$ of 0.992, an RMSE of 0.155 mm, and an MAE of 0.116 mm. With 98.96% of prediction errors falling within the 0.5 mm tolerance, the proposed model fully meets the precision requirements for industrial production. Finally, the framework demonstrates its spatiotemporal capabilities by effectively tracking wire bow variations across different positions (head, middle, and tail) of the wire web. Moreover, SHAP analysis quantitatively confirms that the torques of both the feed and spindle motors are the dominant driving factors, providing theoretical guidance for process parameter optimization.

Despite the promising results in real-time monitoring, this study has certain limitations that outline critical directions for future research. Firstly, the current dataset relies on a single machine over a limited one-month timeframe with constant ingot specifications. Consequently, the model’s external validity across different months or factories, along with the specific impacts of geometric and material variations, remains unverified. Future work will expand the dataset to encompass multi-variable production scenarios, including diverse ingot sizes, materials, extended timeframes, and multiple machines. This will capture their coupling effects and construct a more universal model with validated industrial applicability. Secondly, the current study primarily focuses on wire bow estimation under stable production conditions; thus, model performance under extreme or abnormal scenarios, such as the period immediately preceding a wire breakage, has not yet been investigated. Future research will focus on developing wire bow-based early-warning algorithms for wire breakage. This transition will enable the system to alert operators of excessive wire bow trends in advance, facilitating proactive intervention to minimize material yield loss and production interruptions. Furthermore, while this study establishes a robust predictive framework, its real-time output provides a critical feedback variable for plant-level closed-loop control. Based on the proposed deployment architecture shown in Figure 6, subsequent research will explore automated process modifications, specifically focusing on dynamically adjusting the wire feeding speed and tension to control and modify the wire bow in real time.

## Figures and Tables

**Table 1:** Summary of core mathematical formulations for the six ML models.

Model	Prediction Function
SVR	$\hat{y}=\sum_{i=1}^{n}\left(\alpha_{i}-\alpha_{i}^{*}\right)K\left(x_{i},x\right)+b$
MLP	$\hat{y}=f_{out}\left(W^{\left(2\right)}\cdot g\left(W^{\left(1\right)}x+b^{\left(1\right)}\right)+b^{\left(2\right)}\right)$
DTR	$\hat{y}=\sum_{m=1}^{M}{\hat{c}}_{m}I\left(x\in R_{m}\right)$
RF	$\hat{y}=\frac{1}{T}\sum_{t=1}^{T}h_{t}\left(x\right)$
GB	$\hat{y}=F_{0}\left(x\right)+\sum_{m=1}^{M}\nu \cdot h_{m}\left(x\right)$
XGBoost	$\hat{y}=\sum_{k=1}^{K}f_{k}\left(x\right)$

**Table 2:** Features for wire bow prediction.

Feature Name	Unit	Description	Range	Resolution
Wire Speed	m/s	Tangential speed of the wire web driven by the main spindle rotation.	13~38	±0.1
Feed Relative Position	mm	Relative vertical distance of the ingot from the starting height during the feed process.	0~217	±0.1
Feed Speed	mm/min	Downward feed rate of the ingot (distance moved per minute).	0~3	±0.01
Feed Motor Torque	N·m	Output torque of the motor driving the feeding mechanism.	0~0.463	±0.001
Right Spindle Motor Torque	N·m	Output torque of the right-side motor driving the wire web rotation.	5.67~58.49	±0.01
Left Spindle Motor Torque	N·m	Output torque of the left-side motor driving the wire web rotation.	5.66~58.39	±0.01
Left Tension Motor Position	°	Angular position of the supply-side tension motor, used to regulate wire tension.	0~0.68	±0.01
Cutting Fluid Temperature	°C	Temperature of the cutting fluid entering the cutting chamber.	18.7~21.4	±0.1
Wire bow	mm	Vertical displacement height of the wire web.	0~12	±0.001

**Table 3:** Hyperparameter search space and optimization results for the prediction model.

Hyperparameter	Candidate Values	Description	Stage I	Stage II
n_estimators	[50, 100, 200, 300]	Number of boosting trees.	50	46
max_depth	[3, 5, 7, 9]	Maximum tree depth.	7	7
learning_rate	[0.01, 0.05, 0.1]	Learning rate (controls contribution of each tree).	0.05	0.043
subsample	[0.6, 0.8, 0.9, 1.0]	Subsample ratio of instances (increases diversity).	0.8	0.782
colsample_bytree	[0.6, 0.8, 0.9, 1.0]	Subsample ratio of features (prevents overfitting).	0.8	0.844
reg_alpha	[0, 0.1, 0.5, 1]	L1 regularization term.	0	0.016
reg_lambda	[0, 0.1, 1, 5, 10]	L2 regularization term.	0	0.003

**Table 4:** Hyperparameter settings of the comparison models.

Model	Hyperparameter
SVR	kernel = `rbf’, C=1.0, ϵ=0.1, γ = `scale’
DTR	min_samples_split = 2, min_samples_leaf = 1, max_depth = None
RF	n_estimators = 143, max_depth = None, max_features = `auto’
GB	n_estimators = 56, learning_rate = 0.193, max_depth = 5
MLP	hidden_layer_sizes = (128, 64, 32), activation = `relu’, solver = `adam’, α=0.0001, batch_size = `auto’, learning_rate = `constant’, n_iter_no_change = 10

**Table 5:** Model hyperparameters used for modeling in various positions.

Hyperparameter	Head	Middle	Tail
n_estimators	139	101	145
max_depth	9	7	9
learning_rate	0.071	0.068	0.104
subsample	0.784	0.813	0.728
colsample_bytree	0.964	0.943	0.914
reg_alpha	0.002	0.006	0.078
reg_lambda	0.038	0.006	0.522

## Data Availability

The raw data supporting the conclusions of this article will be made available by the authors on request.

## Abbreviations

The following abbreviations are used in this manuscript:

MWS	Multi-Wire Saw
XGBoost	eXtreme Gradient Boosting
DTR	Decision Tree Regression
SVR	Support Vector Regression
GB	Gradient Boosting
RF	Random Forest
SHAP	SHapley Additive exPlanations
MAE	Mean Absolute Error
MSE	Mean Squared Error
RMSE	Root Mean Squared Error
MAPE	Mean Absolute Percentage Error
